# Supplementary material for: Strong enhancement of photoresponsivity with shrinking the electrodes spacing in few layer GaSe photodetectors
Source: Sci Rep. 2015 Jan 30;5:8130. doi: 10.1038/srep08130 (PMC4311250; doi:10.1038/srep08130)
Supplement: Supplementary Information [file srep08130-s1.pdf]

Supplementary information for strong enhancement of photoresponsivity with shrinking the electrodes spacing in few layer GaSe photodetectors

Yufei Cao[1†], Kaiming Cai[1†], Pingan Hu[2], Lixia Zhao[3], Tengfei Yan[1], Wengang Luo[1], Xinhui Zhang[1], Xiaoguang Wu[1], Kaiyou Wang[1*] and Houzhi Zheng[1]

[1.] SKLSM, Institute of Semiconductors, Chinese Academy of Science, P. O. Box 912, Beijing 100083, P. R. China.

[2.] KLMM, Harbin Institute of Technology, No. 2 YiKuang Street, Harbin, 150080, P.R. China.

[3.] Semiconductor Lighting Technology Research and Development Center, Institute of Semiconductors, Chinese Academy of Sciences, Beijing, 100083, P. R. China.

[†]These authors contributed equally to this work.
[*]Correspondence should be addressed to: kywang@semi.ac.cn

DEVICE MOBILITY MEASUREMENTS

The top contacted device was characterized first in the dark state by applying a constant drain-source voltage V_{DS} = 10 V and sweeping the back-gate voltage V_{BG} [Figure S1]. The typical p type Field Effect transistor behavior was observed. The effective field-effect mobility of the device was estimated from the back gate sweep using the equation $\mu = (\mathrm{d}I_{DS}/\mathrm{d}V_{BG}) \times (l/WC_iV_{DS})$, where l = 1 μm is the channel length, W = 5 μm is the channel width, and C_i = 1.3×10^{-4} Fm^{-2} is the back gate capacitance ($C_i = \varepsilon_0\varepsilon_r/d$, ε_r = 3.9, d = 300 nm). For the device shown in Figure 4a in the manuscript, the field-effect mobility $\mu = 5\times10^{-3}$ cm^2 V^{-1}s^{-1} was obtained.

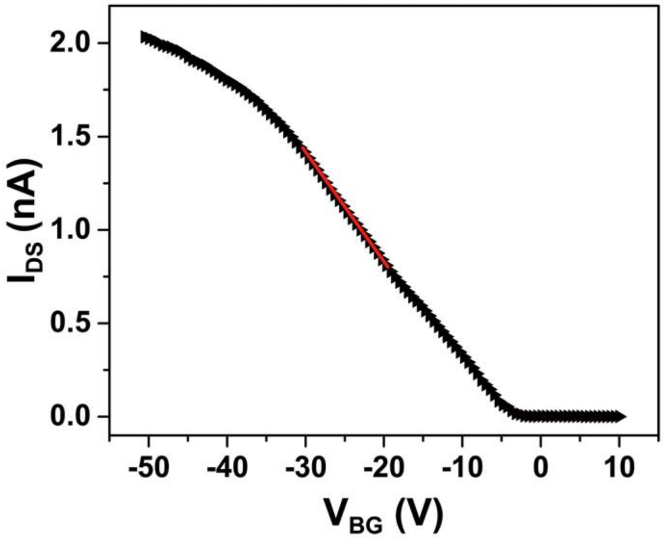

Figure S1. Gating characteristics of the nanosheet GaSe transistor presented in the main manuscript. Room-temperature transfer characteristic of the nanosheet GaSe phototransistor presented in the main manuscript. I_{DS} - V_{BG} sweep is performed at V_{DS} =10 V in the dark state.

AFM RESULTS OF GASE NANOSHEET

The schematic crystal structure was shown in Figure S2c. The spacing distance between the neighbor layers is about 0.90 nm, which was confirmed by the AFM measurements based on the mechanically exfoliated monolayer GaSe [Figure S2]. However, the monolayer GaSe devices show very large resistance which is beyond the measurement accuracy of our instruments. The bad electronic property may be because of easy oxidation of GaSe during the fabrication processing.

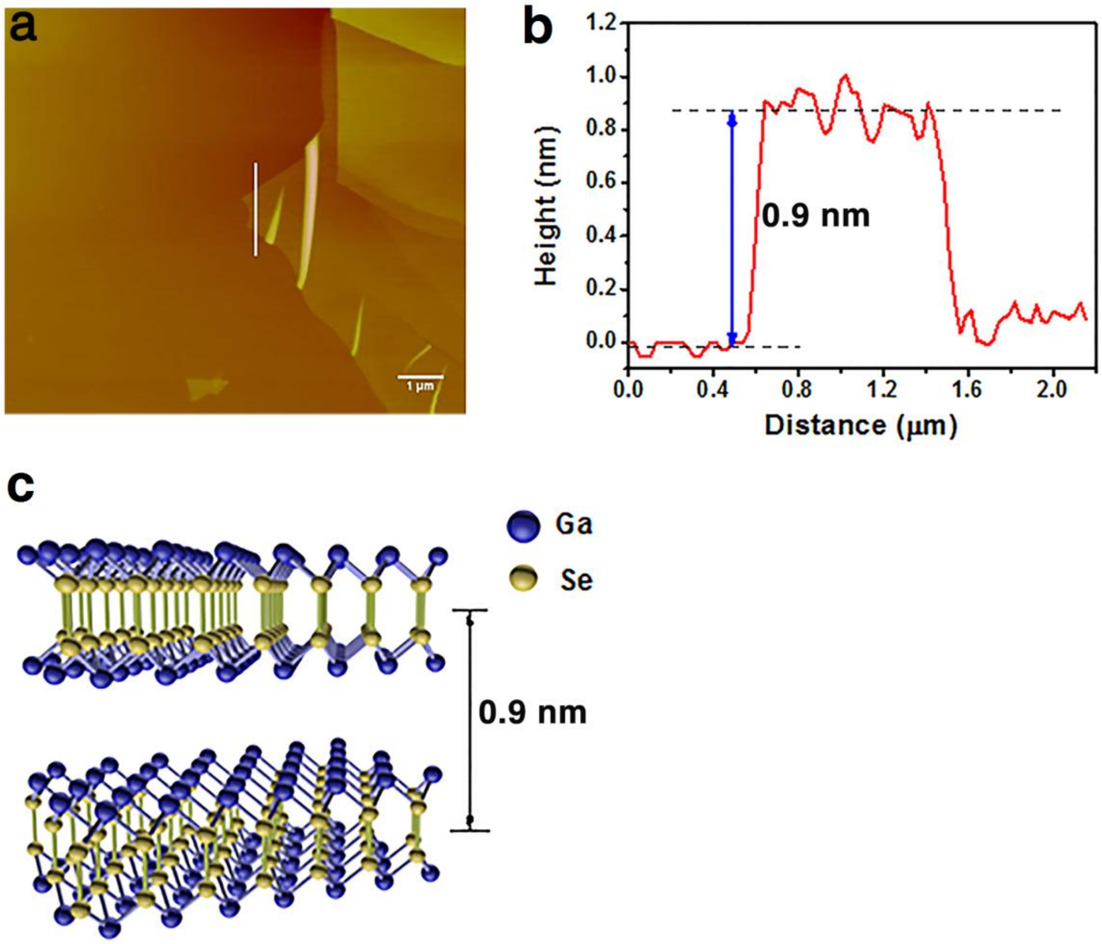

Figure S2. The thickness of the monolayer GaSe measured as 0.9 nm. a, AFM imaging of a few-layer GaSe flake on a silicon substrate with a 300 nm thick oxide layer; **b**, Plot along the white line in a determines the thinnest part; **c**, Three dimensional structure of GaSe, the thickness of the single layer is 0.9 nm.

PHOTOCURRENT AND PHOTORESPONSIVITY RELATIONSHIP WITH PHOTOINTENSITY AT DIFFERENT VOLTAGE.

With the bias voltage above 2 V for the bottom contacted photodetector with l =1 μm, the photocurrent first sharply increases with increasing the light intensity and then the photocurrent shows very slow increase when the light intensity is large enough (Figure S3a). Since the photoresponsivity is proportional to the ratio of photocurrent and the light intensity, thus it shows a reverse result (Figure S3b), which is very similar to that shown in Figure 4d. At a fixed light intensity, both the photocurrent and photoresponsivity increase dramatically at low voltages and then slower down and shows a trend of saturation. Considering the low dark current and large

photoresponse, the distance dependent of the photoresponsivity measured at 8 V was plotted in the manuscript (Figure 2b).

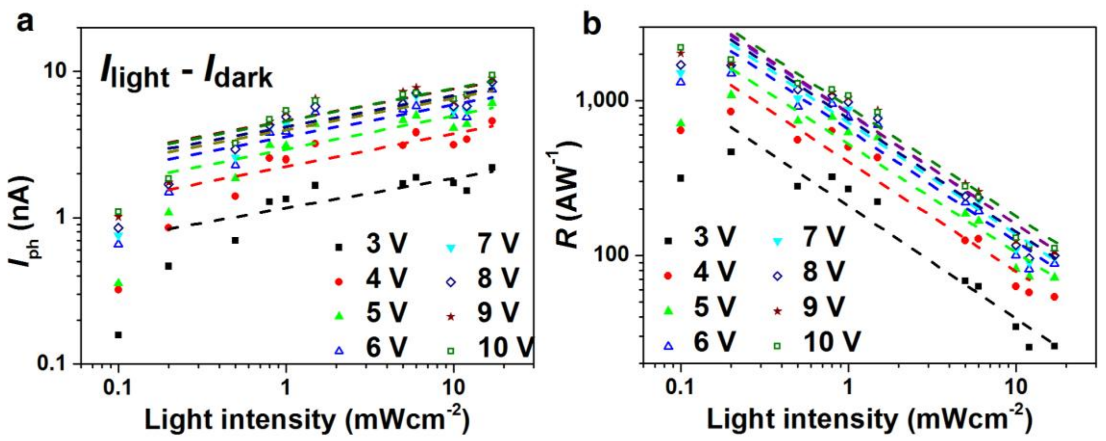

Figure S3. Photocurrent and photoresponsivity with varying incident light power at different bias voltages. a, I_{ph} relationship with light intensity at different bias voltages V_{DS}. **b**, Photoresponsivity relationship with light intensity at different V_{DS}.

PHOTORESPONSIVITY DEPENDENCE OF DISTANCE AND CONTACTS

To demonstrate that concept related to the transport of the photo generated carriers in a metal-semiconductor-metal (MSM) photodetector, a numerical model was developed. The photogenerated electrons diffusive to the interface between the GaSe and the metal contact with higher electrical potential (using $X = l$ at forward bias for example), and then the electrons have the same possibility to pass through the interface and enter into the metal contact. For clarity and simplicity, the depletion region is equivalent to an infinitely thin Schottky barrier at $X = 0$ or $X = l$ depending on the bias direction (take forward namely $X = l$ at positive voltage side as example in this paper).

We firstly discuss the top contacted device. To simplify, considering the device as a one dimensional chain, the number of the photogenerated electrons per second at arbitrary position $X = x$ between the two electrodes is $N(x)$ under global illumination (Figure S4), where the distribution of $N(x)$ will according to the Gaussian function. Then the number of the photogenerated electrons per second in the whole device is $N = N(x)l$.

Under forward bias, the photogenerated electrons will drift to $X = l$ side. During this process, the electrons will be partially recombined. The remaining photogenerated electrons will be exponentially decrease with the travel distance, which can be written down as $N(x)\exp\left[-\left(x'-x\right)/v\tau\right]$, where x' is the arbitrary position at the right side of position x, v is the electron diffusive velocity and τ is the lifetime of the electrons. The electrons generated at position $X = x$ need time $t = (l-x)/v$ to drift from *x* to *l*, then the remaining number of the electrons transport from *x* to electrode (*x* = *l*) is:

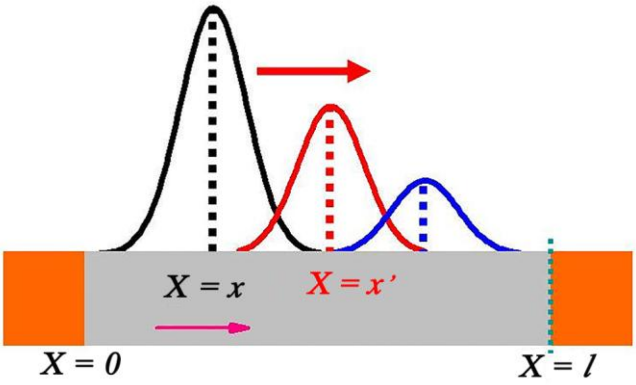

Figure S4. The schematic of the drifting of the photogenerated electrons at position $X = x$ for top contacted device under forward bias.

$$N_0\left(x\right)=N(x\rightarrow l)\approx N\left(x\right)\exp\left[-(l-x)/v\tau\right]\approx N\left(x\right)\exp\left[-(l-x)/L_{\mathrm{D}}\right] \qquad (1)$$

where $L_{\mathrm{D}} = v\tau$ is the diffusion length. Under forward electrical bias, the number of the electrons reached to $X = l$ (the high electrical potential) under global illumination can be written down as: $N = \int_0^L \left(\sigma W\right)\exp\left[-(l-x)/L_{\mathrm{D}}\right]\mathrm{d}x$, where σ is the number of the photogenerated electrons per square meter, *W* is the width of the device. And the photocurrent is proportional to the number of the total electrons per second received at $X = l$, which can be written down as $I_{\mathrm{ph}} = cN = c\int_0^L \left(\sigma W\right)\exp\left[-(l-x)/L_{\mathrm{D}}\right]\mathrm{d}x$, c is a constant as the proportionality of the electrons entering the metal contact at the interface. Then the photocurrent is $I_{\mathrm{ph}} = c\sigma W L_{\mathrm{D}}\left[1-\exp\left(-l/L_{\mathrm{D}}\right)\right] = C\left[1-\exp\left(-l/L_{\mathrm{D}}\right)\right]$, where $C = c\sigma W L_{\mathrm{D}}$.

$I_{\mathrm{ph}} = c\sigma W L_{\mathrm{D}}$ can be obtained in the limit of $l >> L_{\mathrm{D}}$, while $I_{\mathrm{ph}} = c\sigma W l$ can be obtained in the limit of $l << L_{\mathrm{D}}$. Then the corresponding photoresponsivity can be written down as:

$$R = I_{\mathrm{ph}} / P_{\mathrm{light}} = \frac{c\sigma L_{\mathrm{D}}}{L_{\mathrm{intensity}} l}\left[1-\exp\left(-l/L_{\mathrm{D}}\right)\right] = C_0\left[1-\exp\left(-l/L_{\mathrm{D}}\right)\right]/l \tag{2}$$

where $L_{\mathrm{intensity}}$ is the light intensity and $C_0 = c\sigma L_{\mathrm{D}} / L_{\mathrm{intensity}}$. The spacing distance dependence of the photoresponsivity for the top contacted devices can be well fitted using Equation (2), and the fitted diffusive length of $L_{\mathrm{D}} = 170$ nm was obtained.

Considering the photodetectors with bottom contacted electrodes, except for the region from $X = 0$ to $X = l$, the regions of GaSe nanosheet directly on top of the contacts also contribute to the photocurrent under global illumination. The region from X = 0 to X = l, it is the same with the former analysis. Under forward bias, the photogenerated electrons in the left contact region (from $X = -L_{\mathrm{l}}$ to $X = 0$) have to diffusive to the right side and then enter into the metal contact below, which can be described similarly as the above formula. However, the photoexcited electrons in right contact side will have vertical rather than planar transport and then enter into the

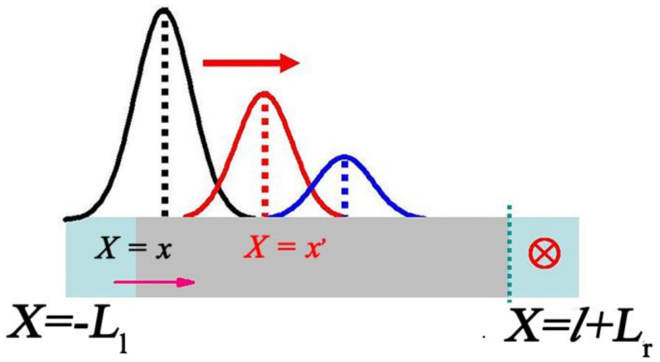

Figure S5. The Schematic of the drifting of the photogenerated electrons at position $X = x$ and also the electrons in the right contact region for bottom contacted device under forward bias.

metal contact below, where the electrons with vertical transport could have different diffusion length L'_{D} and the probability c' entering into the metal contact below

(Figure 5S). Thus the photocurrent is the sum of the planar (the left contact region and the region between the two electrodes) and vertical contributions (right contact region), which can be written down as:

$$\begin{aligned} I_{\mathrm{ph}} &= c\int_{-L_{\mathrm{l}}}^{l} (\sigma W)\exp\left[-(l-x)/L_{\mathrm{D}}\right]\mathrm{d}x + c'\int_{0}^{d}(\sigma W L_{\mathrm{r}})\exp\left[-(d-x)/L'_{\mathrm{D}}\right]\mathrm{d}x \\ &= c\sigma W L_{\mathrm{D}}\left\{1-\exp\left[-(l+L_{\mathrm{l}})/L_{\mathrm{D}}\right]\right\} + c'\sigma W L_{\mathrm{r}} L'_{\mathrm{D}}\left[1-\exp(-d/L'_{\mathrm{D}})\right] \end{aligned} \tag{3}$$

where L_{l} is the width of left contact, c' is the probability of vertical transport electrons enter into the metal contact, L_{r} is the width of the right contact, d is the thickness of the GaSe layer and L'_{D} is the diffusion length. In the above equation, the first term in right hand side describes the photocurrent contribution from the left contact region and the device region between the two electrodes and the second term describes the photocurrent contribution from the right contact region. Only the device area between the two electrodes was counted for calculation of the photoresponsivity in the top contacted device. Thus the photoresponsivity for the bottom contacted device from Equation (3) can be written down as:

$$\begin{aligned} R = I_{\mathrm{ph}}/P_{\mathrm{light}} &= \frac{c\sigma L_{\mathrm{D}}}{L_{\mathrm{intensity}} l}\left\{1-\exp\left[-(l+L_{\mathrm{l}})/L_{\mathrm{D}}\right]\right\} + \frac{c'\sigma L_{\mathrm{r}} L'_{\mathrm{D}}}{L_{\mathrm{intensity}} d l}\left[1-\exp(-d/L'_{\mathrm{D}})\right] \\ &= C_0\left\{1-\exp\left[-(l+L_{\mathrm{l}})/L_{\mathrm{D}}\right]\right\}/l + C_1\left[1-\exp(-d/L'_{\mathrm{D}})\right]/l \end{aligned} \tag{4}$$

where $C_1 = c'\sigma L_{\mathrm{r}} L_{\mathrm{D}}/dL_{\mathrm{intensity}}$. Using L_{D} = 170 nm and the normalized contacts width 700 nm, where the experimental contacts width typically varied between 600 to 800 nm, the distance dependence of the photoresponsivity for the bottom contacted devices can be well fitted using Equation (4) for the bottom contacted devices. The good agreement between the experimental results and the theoretical fittings suggests that our model can well describe the underlying physics of the photodetectors. This model could also be used to explain the photoresponsivity in any two dimensional materials based MSM photodetectors.

SOURCE-DRAIN CURRENT DEPENDENCE OF BIAS VOLTAGE IN DEVICE WITH SINGLE LAYER GTAPHENE ELECTRODES

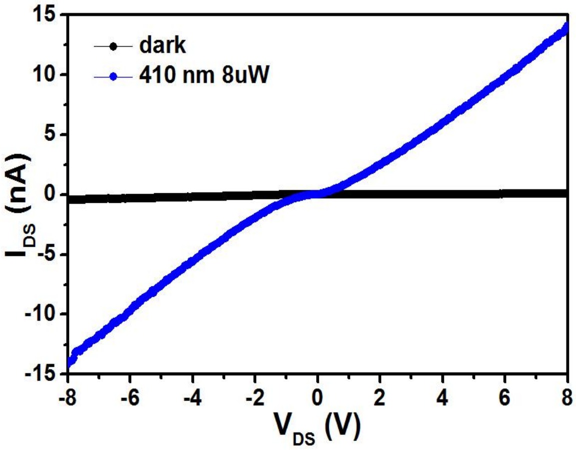

Figure S6. Source-drain current I_{DS} dependence of the bias voltage V_{DS} in the device with single layer graphene electrodes presented in main manuscript (inset of Fig. 4d). The black line is the plot of I_{DS}-V_{DS} with no light irradiation and red line is the plot under irradiation at wavelength of 410 nm. The graphene-GaSe-graphene device shows a low dark current and weak Schottky barrier.
